# Electrochemical Sensor Based on a Carbon Veil Modified by Phytosynthesized Gold Nanoparticles for Determination of Ascorbic Acid

**DOI:** 10.3390/s20061800

**Published:** 2020-03-24

**Authors:** Khiena Z. Brainina, Maria A. Bukharinova, Natalia Yu. Stozhko, Sergey V. Sokolkov, Aleksey V. Tarasov, Marina B. Vidrevich

**Affiliations:** 1 Department of Physics and Chemistry, Research and Innovation Center of Sensor Technologies, Ural State University of Economics, 8 Marta St., 62, 620144 Yekaterinburg, Russia; baz@usue.ru (K.Z.B.); m.a.buharinova@usue.ru (M.A.B.); ssokolkov@yandex.ru (S.V.S.); tarasov_a.v@bk.ru (A.V.T.); mbv@usue.ru (M.B.V.); 2 Department of Analytical Chemistry, Ural Federal University, Mira St. 19, 620002 Yekaterinburg, Russia

**Keywords:** gold nanoparticles, green (phyto) synthesis, carbon veil, electrochemical sensor, ascorbic acid

## Abstract

An original voltammetric sensor (Au-gr/CVE) based on a carbon veil (CV) and phytosynthesized gold nanoparticles (Au-gr) was developed for ascorbic acid (AA) determination. Extract from strawberry leaves was used as source of antioxidants (reducers) for Au-gr phytosynthesis. The sensor was characterized by scanning electron microscopy, energy-dispersive X-ray spectroscopy and electrochemical methods. Optimal parameters of AA determination were chosen. The sensor exhibits a linear response to AA in a wide concentration range (1 μM–5.75 mM) and a limit of detection of 0.05 μM. The developed sensor demonstrated a high intra-day repeatability of 1 μM AA response (RSD = 1.4%) and its stability during six weeks, selectivity of AA determination toward glucose, sucrose, fructose, citric, tartaric and malic acids. The proposed sensor based on Au-gr provides a higher sensitivity and a lower limit of AA detection in comparison with the sensor based on gold nanoparticles synthesized by the Turkevich method. The sensor was successfully applied for the determination of AA content in fruit juices without samples preparation. The recovery of 99%–111% and RSD no more than 6.8% confirm the good reproducibility of the juice analysis results. A good agreement with the potentiometric titration data was obtained. A correlation (r = 0.9867) between the results of AA determination obtained on the developed sensor and integral antioxidant activity of fruit juices was observed.

## 1. Introduction

L-ascorbic acid (AA) (vitamin C) is a water-soluble vitamin with powerful antioxidant properties, which is actively involved in the biochemical processes of the human body. Unlike plants and most animals that have the ability to synthesize AA from glucose, the human body can receive the required amount of AA only from external sources. The physiological level of AA in the body is ensured by its intake only from outside (the use of foods rich in vitamin C, various food additives and pharmaceuticals). Large quantities of AA are contained in fresh fruits, vegetables, berries, and juices [1]. It is added to certain foods and drinks to give them antioxidant properties and to prevent color and taste changes. Taking into consideration the nutritional value and therapeutic AA properties, monitoring of AA content should be recognized as an important and relevant task for assessing the quality of finished food products, raw materials and a number of other substances.

A variety of analytical methods are used to determine AA: titrimetric [2,3], spectrophotometric [2,3,4], chromatographic [5,6,7,8], fluorimetric [9], chemiluminescent and electrochemical methods [10,11,12]. Traditional methods of redox titration do not allow a quantitative assessment of the content of ascorbic acid in stained samples. These methods are low-selective and time consuming. UV-spectrophotometric methods are not suitable for the analysis of complex samples, since other organic compounds are also capable of absorbing ultraviolet radiation. Chromatographic methods, despite the selective detection of many compounds, are quite laborious and time-consuming. Advantages of electrochemical methods for determining AA include the lack of complex sample preparation, high sensitivity and selectivity, quick response, simple instrumentation and its maintenance, which makes it possible to conduct on-site and in-situ measurements. All this indicates the preference for using electrochemical methods of analysis over others.

The main part of an electrochemical system which forms an analytical signal is the electrode/sensor. As a rule, an electrode modified, in one way or another, serves as a sensor. Currently, carbon materials are widely used in sensor technologies: graphene [13], graphene oxide [14,15], carbon nanotubes [16] to increase the sensor surface, its electrical conductivity and catalytic activity. Unique properties (catalytic, electronic, magnetic, high surface to volume ratio) make nanoparticles highly effective components of sensors [17]. To determine AA, nanosized oxides [14] and metals [14,16,18,19] are used. Nanoparticles accelerate electron transfer and reduce overvoltage of electrochemical process [20,21,22,23]. Often, nanomaterials are combined with conductive polymer films to ensure selective measurements and fixing nanoparticles on the electrode surface [14,19,24]. It is worth noting that preparing complex, multi-component modifiers requires quite a lot of time, the use of harmful solvents and complex tools [14,16]. In this regard, it is of current interest to further search and develop new highly sensitive and selective electrochemical sensors for AA determination using modern materials and simple, in particular, “green” technologies. Phytosynthesis is an excellent alternative to chemical and physical methods of the nanoparticles’ synthesis, because it is safe, eco-friendly, express, low-energy consuming, and cost-effective. The synthesis process does not require the application of toxic substances, solvents or surfactants. The role of reducing agents and suspension stabilizers is performed by substances contained in plants. The nanoparticles thus obtained are more stable and less toxic than those obtained by the traditional citrate method [25]. In addition, “green” nanoparticles are biocompatible and exhibit anti-inflammatory, antimicrobial and antifungal activity [26,27]. A promising material for application in electrochemical sensors is a flexible electrically conductive carbon veil, with a developed surface. The carbon veil is used in capacitors [28], batteries [29,30,31], corrosion barriers [32] and as an electrothermal material [33,34]. Only a few examples of carbon veil use in electrochemical sensors are known, e.g., for the determination of nitrite ions [35,36], dopamine [37], adenosine-tri-phosphate [38], trace amounts of silver [39]. Thus, a transducer-carbon veil, modified with nanoparticles, may serve as a good sensor.

The aims of this research were (i) to study the relationship between electrochemical and morphological properties of electrodes modified with gold nanoparticles obtained by phytosynthesis using extracts from strawberry leaves, (ii) creation of a voltammetric sensor based on a carbon veil and gold nanoparticles obtained by green synthesis, and (iii) algorithm development for AA determination with the use of a new sensor.

## 2. Materials and Methods

### 2.1. Reagents 

The following chemically pure reagents were used: ascorbic acid (Sigma-Aldrich Co, St. Louis, MO, USA), Na_2_HPO_4_·12H_2_O and 2,6-dichlorophenolindophenol sodium salt hydrate (CJSC Vekton, St. Petersburg, Russia); KH_2_PO_4_, malik acid and glucose (NevaReaktiv Ltd., St. Petersburg, Russia), sucrose, fructose (JSC LenReactive, St. Petersburg, Russia), HAuCl_4_ (RPE Tomanalyt Ltd., Tomsk, Russia), sodium citrate, citric acid and NaOH (JSC ChemReactivSnab, Ufa, Russia), K_4_[Fe(CN)_6_]·3H_2_O (JSC Reachim Ltd., Moscow, Russia), tartaric acid (Merck KGaA, Darmstadt, Germany), Cementit universal (Merz+Benteli AG, Niederwangen, Switzerland), acetone (Ecos-1, Moscow, Russia), HCl (SigmaTec, Khimki, Russia). All chemicals were used without further purification. Deionized water with a resistivity of 18 MΩ cm was used as the solvent.

### 2.2. Instruments

To obtain gold nanoparticles, a magnetic stirrer with controlled heating RCT basic (IKA-Werke, Staufen, Germany) was used. MIKRO 120 centrifuge (Andreas Hettich GmbH, Tuttlingen, Germany) was used to wash the nanoparticles. A laminator (LM-260iD (Rayson Electrical MFG., Ltd., Foshan, GuangDong, China) was used for manufacturing carbon veil electrodes. Deionized water with a resistivity of 18 MΩ cm was obtained on an Akvalab-UVOI-MF-1812 installation (JSC RPC Mediana-filter, Moscow, Russia). Scanning electron microscopy (SEM) measurements were performed on a Scios 2 microscope (Thermo Fisher Scientific, Pardubice, The Czech Republic) equipped with a Ultim Max detector (Oxford Instruments plc., Abingdon, UK) to perform an analysis of energy-dispersive X-ray spectroscopy (EDS). Electrochemical (voltammetric and amperometric) studies were conducted on an IVA-5 analyzer (IVA Ltd., Ekaterinburg, Russia). Potentiometric titration was carried out on pH/ions meter TA-ION (RPE Tomanalyt Ltd., Tomsk, Russia).

### 2.3. Procedures

#### 2.3.1. Synthesis and Characterization of Gold Nanoparticles 

Two methods of gold nanoparticle’s synthesis were used: Turkevich method [40] and “green” synthesis [25]. In the first case, 750 μL of freshly prepared 0.1 M sodium citrate was added to 15 mL of a boiling 1 mM HAuCl_4_ solution and nanoparticles (Au-cit) were obtained. According to the “green” synthesis procedure, plant extract prepared as described in [41] was used as a reducing agent and a stabilizer of nanoparticles. To 5 mL of boiling 1 mM HAuCl_4_ solution, 1 mL of freshly prepared extract from strawberry leaves was added with vigorous stirring (pH 11). The change in color of the reaction mixture from pale yellow to burgundy red stable for 2 min indicated Au-gr nanoparticles formation. The resulting sol was cooled to room temperature with constant stirring. After this, the sol was centrifuged at 14,000 rpm for 10 min, and the precipitate was washed with deionized water to eliminate excess of unreacted plant extract. The washing procedure was repeated twice. Nanoparticles were separated from the supernatant and resuspended in the initial volume of deionized water. The resulting sol was stored at +4 °C for future application.

Comparison of synthesized Au-gr and Au-cit was carried out basing on the results of electrochemical studies performed in this work and previously published results [25,42].

#### 2.3.2. Manufacturing of the Sensor (Au-gr/CVE)

A carbon veil (CV) with a area density of 30 gm^−2^ (M-Carbo, Russia) was used as a transducer. CV was glued onto a polyethylene terephthalate film 216 × 303 mm^2^ size and 250 μm thick (Fellows, Vietnam) by hot lamination at 150 °C. Carbon veil-coated film was cut into 35 × 3 mm^2^ strips. The middle part of the strip separating working and contact zones was covered with a Cementit-acetone mixture in a ratio of 1:5 by volume. The geometric area of the working zone was 15 mm^2^ (5 × 3 mm). Electrode (CVE) manufactured in this way was modified by drop casting of a gold sol (1 layer = 5 μL) and dried in the air. CVE, modified with Au-gr, was later called a sensor Au-gr/CVE.

#### 2.3.3. Electrochemical Measurements

Electrochemical studies were performed by cyclic and linear sweep voltammetry, as well as chronoamperometry in a three-electrode cell, including a silver-silver chloride reference electrode (Ag/AgCl/KCl, 3.5M) (Gomel, Belarus), a carbon rod as an auxiliary electrode, and working electrodes CVE, Au-gr/CVE and Au-cit/CVE. Working electrodes were washed with deionized water before application.

Cyclic voltammograms of AA were recorded in the potential range from −0.1 V to +1.2 V on CVE and from −0.1 V to +0.8 V on Au-gr/CVE at potential scanning rate 0.05 Vs^−1^. The limitations of the potential scan range for electrodes modified with gold nanoparticles are due to the fact that the gold nanoparticles electrooxidation process starts at 0.9 V.

Cyclic voltammograms 1 mM [Fe(CN)_6_]^4−/3−^ were recorded in the potential range from −0.5 V to +1.0 V at a potential scan rate 0.05 Vs^−1^. 

Linear sweep (LS) voltammograms of AA were recorded at anodic potential scanning in the range from −0.1 V to 0.8 V. The potential scanning rate was varied within 0.05–0.40 Vs^−1^. 

Chronoamperometric measurements were carried out at potential +0.7 V in the solution containing 1.0 mM K_4_[Fe(CN)_6_] + 0.1 M KCl, and at potential +0.45 V in the solution containing 0.1 mM AA.

### 2.4. Statistical Analysis and Data Treatment 

All measurements were carried out 3 times and the results were calculated for a confidence level of 0.95. The results are presented as X ± ΔX, where X is the average value and ΔX is the standard deviation. The recovery of AA was calculated according to IUPAC recommendations [43]. Limits of detection (LOD) and quantification (LOQ) were calculated as 3SD/*b* and 10SD/*b*, respectively, where SD is the standard deviation of the response and *b* is the slope of the calibration graphic. 

*F*- and *t*-tests were used to compare the results of AA determination in the juices obtained on the developed sensor Au-gr/CVE and the reference potentiometric titration method.

## 3. Results

### 3.1. Electrochemical Behavior of Ascorbic Acid 

**Figure 1:**
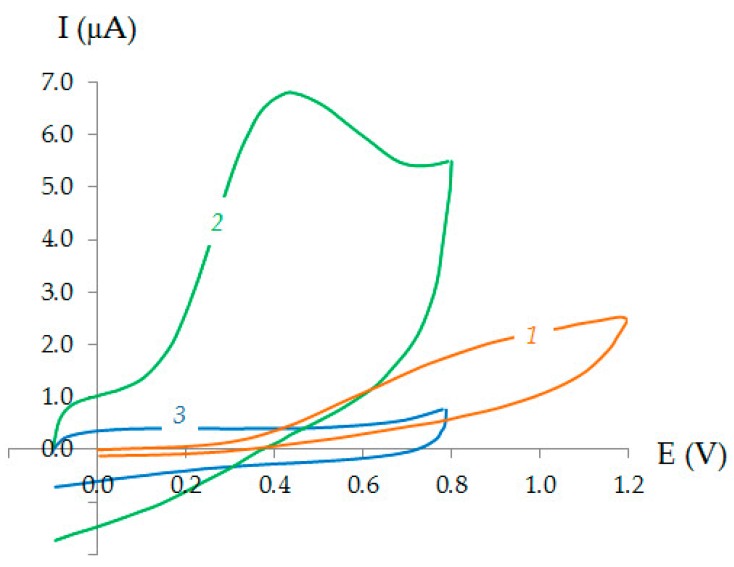
Cyclic voltammograms on CVE (**1**) and Au-gr/CVE (**2**,**3**) in the PBS, pH 7, without (**3**) and in the presence of 0.1 mM AA (**1**,**2**). ν = 0.05 Vs^−1^.

Figure 1 shows cyclic voltammograms on Au-gr/CVE in the PBS, pH 7, not containing and containing 0.1 mM AA. It can be seen from Figure 1 that on the cyclic voltammogram (curve 3) at Au-gr/CVE in PBS, there are no anodic and cathodic signals. The introduction of AA into the background electrolyte led to the appearance of a poorly expressed, small anodic wave of AA on CVE (curve 1) and a clear signal three times larger in size on Au-gr/CVE (curve 2). In this case, the potential of the AA oxidation current on Au-gr/CVE was shifted more than 0.4 V to the cathodic region, compared with that observed on CVE. 

**Figure 2:**
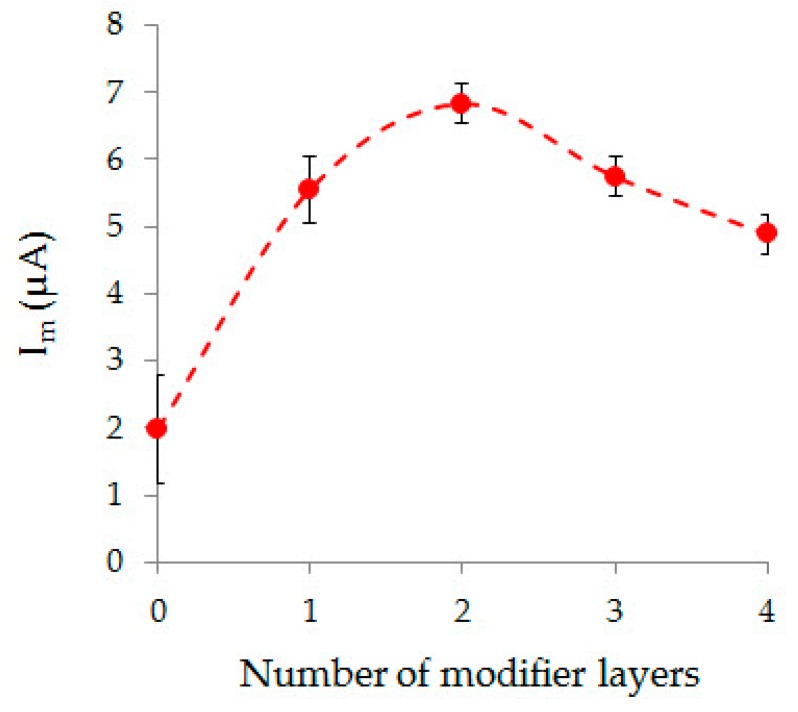
Effect of the modifier layers number on the oxidation current of 0.1 mM AA on Au-gr/CVE in the PBS pH 7.

The dependence of AA oxidation current on Au-gr/CVE on number of Au-gr layers deposited on the CVE surface is shown in Figure 2. It can be seen from Figure 2 that the largest AA current was obtained on CVE modified with two Au-gr layers. Obviously, one Au-gr layer is not enough to obtain the highest current of AA, and the sequential deposition of more than two Au-gr layers leads to the gradual formation of a macro-gold coating on CVE and a decrease of the active gold surface, which causes a decrease of AA current. Therefore, CVE was modified with two Au-gr layers.

### 3.2. Characterization of CVE and Au-gr/CVE 

**Figure 3:**
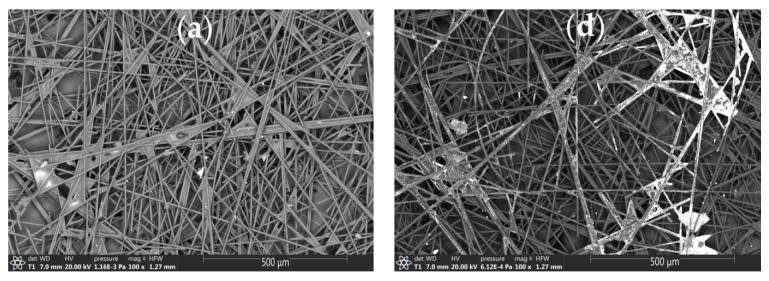

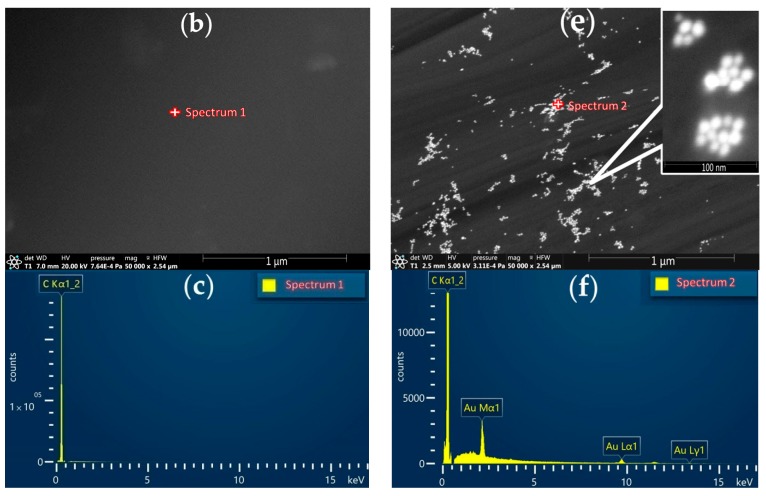
SEM-images of bare CVE (**a**,**b**) and Au-gr/CVE (**d**,**e**) and EDS spectrum of CVE (**c**) and Au-gr/CVE (**f**).

Figure 3 shows micrographs of the CVE and Au-gr/CVE surfaces obtained by SEM. As can be seen from Figure 3a, the CVE surface is randomly interwoven fibers (5 to 10 μm diameter), with a binder in some parts of which. As can be seen from the EDS spectrum, the main element of CVE is carbon (Figure 3c). The Au-gr/CVE surface consists of fibers with a whitish coating (Figure 3d), which, at a higher magnification, are identified as single small bright points 10–15 nm in size and their associates 30–40 nm in size (Figure 3e). Au-gr/CVE EDS spectrum (Figure 3f), recorded in the bright spot, confirms the presence of gold in it.

**Figure 4:**
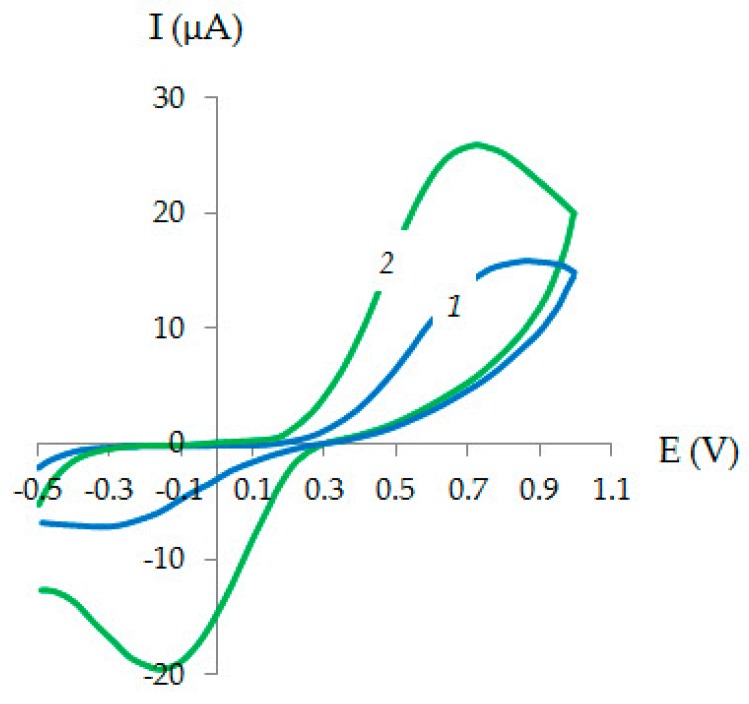
Cyclic voltammograms of 1 mM [Fe(CN)_6_]^4−^ on CVE (**1**) and Au-gr/CVE (**2**) in 0.1 M KCl. *ν* = 0.05 Vs^−1^.

Figure 4 shows cyclic voltammograms on CVE and Au-gr/CVE in 0.1 M KCl containing 1 mM K_4_[Fe(CN)_6_]. An increase of the oxidation-reduction currents K_3_[Fe(CN)_6_]/K_4_[Fe(CN)_6_] on Au-gr/CVE is observed in comparison with CVE. The potential difference between cathodic and anodic peaks is 0.88 V on Au-gr/CVE and 1.19 V on CVE. The ratio of the anodic and cathodic peak currents (I_ma_/I_mc_) is 2.21 for CVE and 1.33 for Au-gr/CVE. A decrease in the potential difference of currents confirms the higher rate of electrochemical processes on Au-gr/CVE compared to CVE.

**Figure 5:**
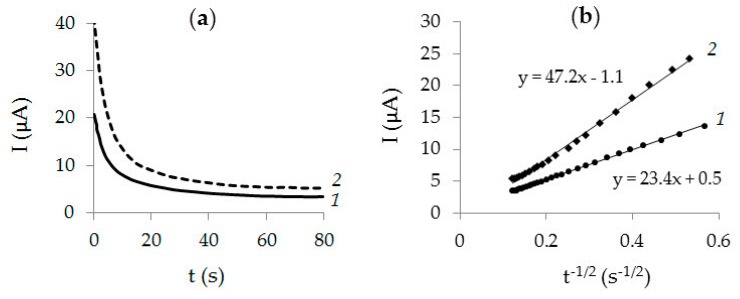
Chronoamperograms obtained on CVE (**1**) and Au-gr/CVE (**2**) in 1.0 mM K_4_[Fe(CN)_6_] + 0.1 M KCl, *E* = 0.7 V (**a**). Dependencies *I* = f (*t*^−1/2^) are obtained from chronoamperograms on the corresponding electrodes (**b**).

Figure 5 shows chronoamperograms of K_4_[Fe(CN)_6_] oxidation at a potential 0.7 V on CVE and Au-gr/CVE (Figure 5a), as well as the dependences I = f (t^−1/2^) (Figure 5b). As can be seen from Figure 5b, the slope of the linear relationship I = f (t^−1/2^) for Au-gr/CVE is two times greater than for CVE. The effective surface area of the electrodes was calculated using Cottrel ecuation [44] and the above-mentioned data. Thus, the area of CVE appeared to be 15.6 mm^2^ and 32 mm^2^ for Au-gr/CVE.

Table 1 presents comparative characteristics of Au-gr and Au-cit nanoparticles based on previously published results (TEM, UV-Vis-spectrophotometry) [25,42] and the LS Voltammetry results obtained in this work.

It can be seen from Table 1 that Au-gr and Au-cit have a spherical shape and a similar average diameter; however, unlike Au-gr, about 10 % of large nanoparticles 38 nm in diameter are found among Au-cit. According to UV-Vis-spectrophotometry, Au-gr nanoparticles are smaller than Au-cit. According to the results of voltammetric studies, Au-gr is more electrochemically active than Au-cit. Thus, at similar values of Au-gr and Au-cit oxidation peak currents in 1 M HCl, Au-gr peak potential is shifted 26 mV to the cathodic region. It is known that with an increased electrochemical activity of gold nanoparticles, the overvoltage of substances electrooxidation decreases on the nanoparticles [22]. Indeed, AA oxidation peak current on Au-gr is detected 22 mV earlier compared to Au-cit.

### 3.3. Electrooxidation of Ascorbic Acid on Au-gr/CVE

**Figure 6:**
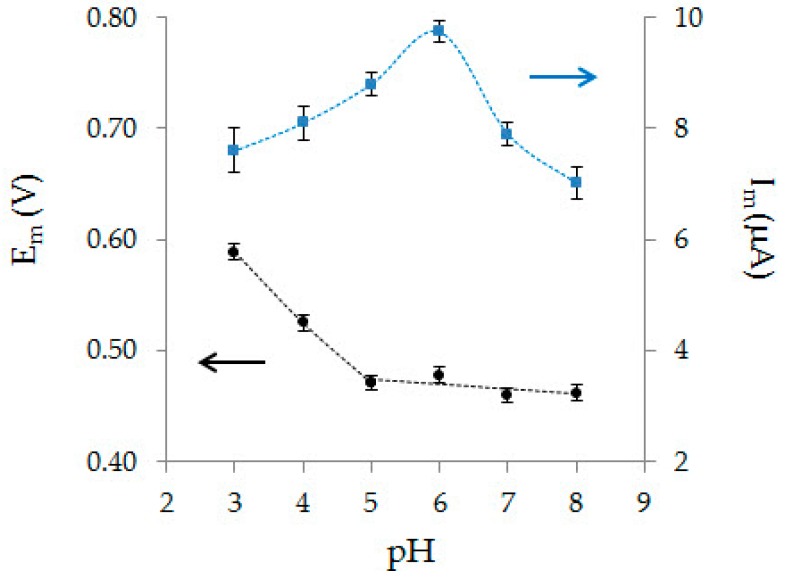
Effect of background electrolyte pH on AA oxidation peak current (*I*_m_) and peak potential (*E*_m_) (0.1 mM) on Au-gr/CVE.

The impact of the background electrolyte pH on voltammetric characteristics of AA oxidation on Au-gr/CVE is shown in Figure 6. With pH an increase from 3 to 5 AA, peak potential sharply shifts to the cathodic side. In the pH range from 5 to 8, AA peak current recorded on Au-gr/CVE remains almost the same. The dependence of the AA peak potential on pH in the range from 3 to 5 is described by Equation (1):

*E*_m_ (V) = (0.7643 ± 0.0458) − (0.0590 ± 0.0030) pH, R^2^ = 0.9976 (1)

The obtained slope value is 0.059 V, which corresponds to the theoretical value [45] for the process in which the same number of protons and electrons takes part. As can be seen from Figure 6, the highest oxidation current of AA is observed at pH 6; therefore PBS with a pH of 6 was used as the background electrolyte for further studies. 

**Figure 7:**
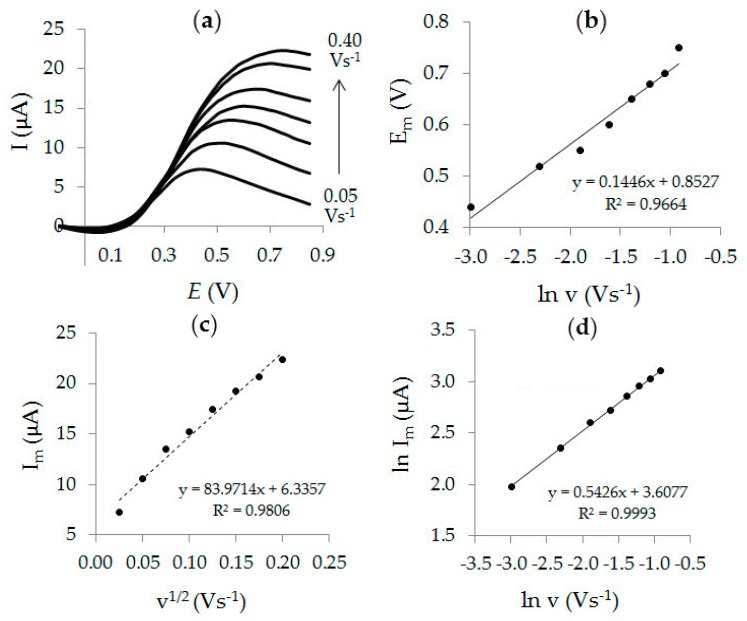
LS voltammograms of 0.1 mM AA on Au-gr/CVE in PBS pH 6 at different potential scan rates (0.05–0.40 Vs^−1^), (**a**) and dependences *E*_m_ = f (ln *ν*) (**b**), *I*_m_ = f (*ν*^1/2^) (**c**), ln *I*_m_ = f (ln *ν*) (**d**).

Figure 7a shows that influence of the potential scan rate in the range from 0.05 to 0.40 Vs^−1^ on the AA current and the oxidation potential. As can be seen from Figure 7c, the AA peak current increases and its potential shifts to an anodic region with an increase in the potential scan rate. The shift of the AA oxidation potential to the anodic region with the scan rate increase (Figure 7b) is characteristic of the irreversible electrode process [45] and can be expressed by the corresponding Equation (2):

E_m_ (V) = (0.853±0.035) + (0.145±0.012) ln ν (Vs^−1^), R^2^ = 0.9664 (2)

The linear dependence of the AA oxidation peak current on the square root of the scan rate (Figure 7c) indicates that the electrochemical process under consideration is diffusion controlled. This conclusion also follows from the dependence of the natural logarithm of the AA peak current on the natural logarithm of the potential scan rate (Figure 7d). The slope of the dependence ln *I*_m_ = f (ln *ν*) is 0.54 and is close to the theoretical value of 0.5, which is characteristic of a diffusion-controlled process [46].

### 3.4. Analytic Characteristics of Au-gr/CVE

**Figure 8:**
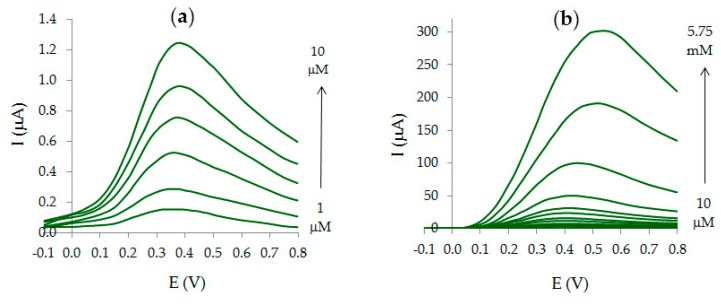

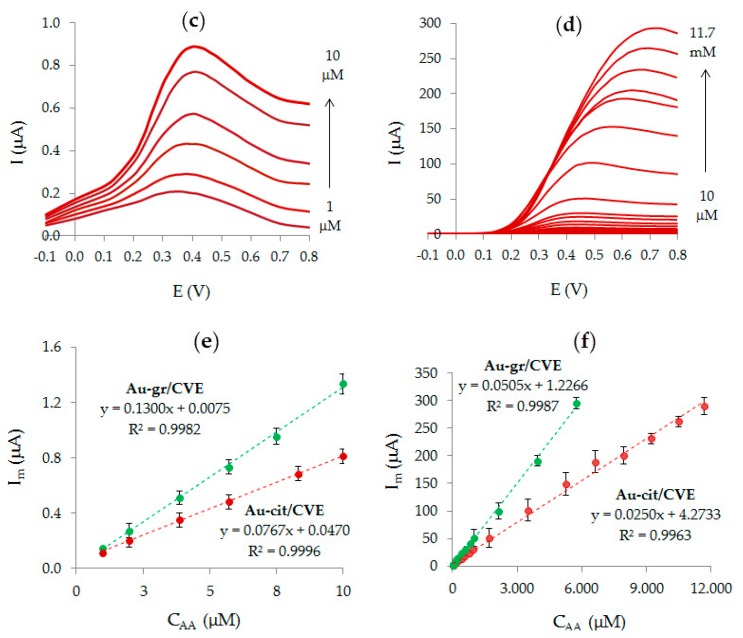
AA voltammograms on Au-gr/CVE (**a**,**b**) and Au-cit/CVE (**c**,**d**) at different AA concentrations and corresponding dependences *I*_m_ vs *C*_AA_ on Au-gr/CVE and Au-cit/CVE (**e**,**f**).

The dependence of the peak current on AA concentration on Au-gr/CVE and Au-cit/CVE is shown in Figure 8. As can be seen from the Figure 8e,f, the slope of the calibration curves for Au-gr/CVE is higher than that for Au-cit/CVE, which testifies the higher sensitivity of Au-gr/CVE (0.13 µAµM^−1^) compared to Au-cit/CVE (0.05 µAµM^−1^). 

LOD and LOQ are 0.05 and 0.15 µM for Au-gr/CVE, 0.20 and 0.60 µM for Au-cit/CVE. Thus, Au-gr/CVE has four times lower LOD and LOQ compared to Au-cit/CVE. The relative standard deviation (RSD) of AA 1 μM response is 1.4% on Au-gr/CVE and 3.6% on Au-cit/CVE. 

The results of the interfering effect studies of a number of compounds that are part of real juice samples [47] on the AA response on Au-gr/CVE are presented in Table 2. It can be seen that a 100-fold excess of glucose, a 500-fold excess of sucrose, a 600-fold excess fructose and citric acid, a 800-fold excess of tartaric and a 1000-fold excess of malic acids do not interfere with AA determination.

**Figure 9:**
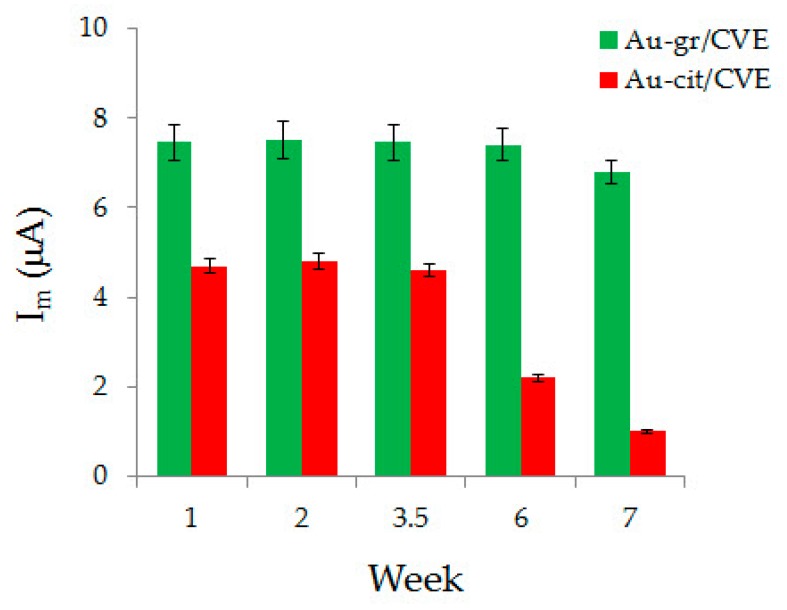
Stability of AA response on Au-gr/CVE and Au-cit/CVE.

0.1 mM AA response on the proposed sensor is stable for 6 weeks, which is shown on the diagram (Figure 9). As can be seen from the diagram, 0.1 mM AA oxidation current on Au-gr/CVE does not change for 6 weeks, but by the 7th week, it is reduced by 8%. On Au-cit/CVE, the 0.1 mM AA response is stable for 3.5 weeks and decreases by almost 50% by the 6th week.

The data presented testify that the proposed Au-gr/CVE provides an improvement in the number of analytical characteristics (sensitivity, LOD, LOQ, inter- and intra-day repeatability) compared with Au-cit/CVE, which, apparently, is due to the higher electrochemical activity of Au-gr compared to Au-cit (Table 1).

Table 3 summarizes the analytical characteristics of various sensors used for AA content determination in foods and pharmaceuticals.

It can be seen from Table 3 that detection limit and linear range, which are characteristic of the developed sensor, are not only comparable, but are also better in comparison with many other modified electrodes.

### 3.5. Determination of Ascorbic Acid in Fruit Juices 

Juice aliquot (0.05–0.40 mL) was placed in an electrochemical cell containing phosphate buffer solution (PBS), pH 6, thoroughly mixed, and LS voltammogram was recorded from 0.0 V to +0.8 V at a scan rate of 0.05 Vs^−1^. Anodic peak current served as the response.

The results of the ascorbic acid content analysis in fruit juices on the proposed sensor are presented in Table 4.

As can be seen from Table 3, recovery (R) is in the range from 99% to 111%, which confirms the correctness of the AA determination results. 

A validation of the results of AA determination in juices on the developed sensor Au-gr/CVE was carried out with respect to the results obtained by the potentiometric titration reference method [57], in which sodium 2,6-dichlorophenolindophenolate solution was used as a titrant.

The comparison of the juices’ analysis results on the proposed Au-gr/CVE sensor and reference method results is shown in Table 5.

As can be seen from Table 5, RSD does not exceed 6.8 % for Au-gr/CVE. The results obtained by the two methods are in good agreement. The values calculated by the *F*-test and *t*-test were smaller than the critical values at P = 0.95, a fact which indicates the absence of a systematic error and the equivalence of the results obtained on the proposed sensor and by the potentiometric titration reference method.

**Figure 10:**
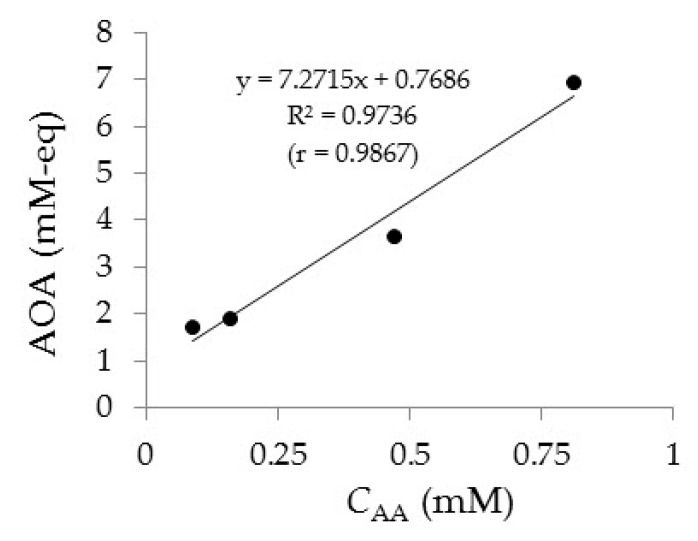
Correlation between AA content and AOA value of fruit juices.

The relationship between AA content in juice samples and their antioxidant activity (AOA) is shown in Figure 10. Assessment of AOA drinks was carried out by the potentiometric method described in [58]. A good correlation is observed between the determination results of AA by the voltammetric method on the Au-gr/CVE and AOA value of the fruit juices samples. The correlation coefficient is 0.9867 (Figure 10).

## 4. Conclusions

A new sensor based on a carbon veil, modified with “green” gold nanoparticles is described in this paper. The sufficiently high conductivity and large active surface of the sensor, application of modern technology of hot lamination and electrochemical registration of the signal provide high analytical characteristics of the determination of ascorbic acid concentration in comparison with the other sensors. The developed sensor is characterized by a low detection limit, a wide linear range, and good measurement’s reproducibility. The high selectivity of the sensor ensured its successful application in a fruit juices analysis without preliminary preparation. The correctness of the juices’ analysis results on the developed sensor is confirmed by satisfactory agreement with the results of the potentiometric titration reference method. A good correlation is shown between ascorbic acid content in fruit juices and their integral antioxidant activity.

## Figures and Tables

**Table 1:** Comparative characteristics of Au-gr and Au-cit nanoparticles.

Method	Parameter	Au-gr	Au-cit
TEM	Shape	Spherical	Spherical
	*d*, nm	14 ± 7 [25]	13 and 38 (up to 10 %) [42]
UV-Vis-spectrophotometry	*d*, nm	10 ± 1 [25]	20 [42]
LS Voltammetry	*I*_m_ (Au), µA	31 ± 5	27 ± 4
	*E*_m_ (Au), V	0.851 ± 0.002	0.877 ± 0.011
	*E*_m_ (AA), V	0.420 ± 0.006	0.442 ± 0.007

*d*—diameter (nm), *I*_m_—oxidation peak current (μA), *E*_m_—peak potential (V).

**Table 2:** Interfering effect of some substances on AA response (AA response at *C*_AA_ = 0.01 mM taken as 100%).

Interfering Substance	Concentration of Interfering Substance (*C*_IS_), mM	*C*_IS_: *C*_AA_	AA Response Change, %
Glucose	1	100	−9.5
Sucrose	5	500	+4.2
Fructose	6	600	+4.8
Citric acid	6	600	−0.5
Tartaric acid	8	800	−1.6
Malic acid	10	1000	−0.5

**Table 3:** Analytical characteristics of AA determination in pharmaceutical tablets, fruit samples, juice and wine on different sensors.

Sensor	LOD, μM	LR, μM	Technique	Object	Ref.
TiO_2_-Au_nps_-MWCNT-DHP/GCE	1.20	5–51	Am	pharmaceutical and orange juice samples	[16]
Au_nps_-PAN/GCE	8.20	10–12000	Am	medicine vitamin C tablets	[19]
Au_nps_-ZnO-PPy-RGO/GCE	0.16	2–950	DPV	vitamin C tablets	[14]
Au_nps_-L-Alanine/GCE	10.00	12–160	CV	–	[18]
Pt-electrode	87.00	310–20000	DPV	fruit juices, wine	[48]
CPE	20.00	70–20000	DPV	–	[48]
PEDOT/GCE	23.30	50–90	SWV	orange and pineapple juices	[49]
SPCE	1360.00	0–10000	CV	packed orange juice sample	[50]
CPE	1.76	10–100	SWV	pharmaceutical tablets	[51]
PoPDoAP/GCE	36.40	100–1000	DPV	vitamin C tablet and orange juices	[52]
Ppy/Au-MA	5.00	10–2200	SWV	lemon juice and celin tablet chewable	[53]
GCE	11.50	8–80	CV, SWV	beverages and fresh edible vegetables	[54]
Fe(III)-Y zeolite/CPE	0.02	0.4–1200	SWV	citrus juices	[55]
CPE	22.10	-	CV	fruit juices	[56]
Au-gr/CVE	0.05	1–5750	AV	fruit juice	[this work]

Am—amperometry, DPV—differential pulse voltammetry, CV—cyclic voltammetry, SWV—square wave voltammetry, AV—anodic voltammetry, MWCNT—multi-walled carbon nanotubes, DHP—dihexadecylphosphate film, CPE—carbon paste electrode, PEDOT—Poly(3,4-Ethylenedioxythiophene), PoPDoAP—poly(o-phenylenediamine-*co*-o-aminophenol), Ppy—polypyrrole, SPCE—screen-printed carbon electrode, Au-MA—gold macro electrode.

**Table 4:** Results determination of AA in fruit juices using the proposed sensor Au-gr/CVE (n = 3, P = 0.95).

Sample	Found in the Sample, mM	Added, mM	Found in the Sample with Additive, mM	Found Additive, mM	R, %
Cherry-apple juice	0.81 ± 0.06	1.94	2.96 ± 0.21	2.15 ± 0.23	111
Apple juice for children	0.47 ± 0.01	0.97	1.43 ± 0.03	0.96 ± 0.02	99
Apple juice	0.16 ± 0.01	0.25	0.418 ± 0.003	0.26 ± 0.01	104
Apple nectar clarified.	0.09 ± 0.01	0.12	0.21 ± 0.02	0.120 ± 0.003	100

**Table 5:** Results of AA determination on the proposed Au-gr/CVE sensor and potentiometric titration in fruit juices (n = 3, P = 0.95).

Samples	Found Using Au-gr/CVE	RSD, %	Found by Potentiometric Titration	RSD, %	*F*-Test	*t*-Test
Cherry-apple juice	0.81 ± 0.06	6.8	0.76 ± 0.07	8.7	1.43	0.67
Apple juice for children	0.47 ± 0.01	2.2	0.42 ± 0.03	7.6	9.00	1.82
Apple juice	0.16 ± 0.01	5.0	0.16 ± 0.01	4.5	1.18	0.49
Apple nectar clarified	0.09 ± 0.01	6.5	0.09 ± 0.01	9.0	1.97	0.19

*F*_crit._ = 19.00 at P = 0.95 и df_1_ = 2, df_2_ = 2; *t*_crit._ = 2.78 at P = 0.95 и df = 4.
